# Improving the Chemical Selectivity of an Electronic Nose to TNT, DNT and RDX Using Machine Learning

**DOI:** 10.3390/s19235207

**Published:** 2019-11-27

**Authors:** Anton Gradišek, Marion van Midden, Matija Koterle, Vid Prezelj, Drago Strle, Bogdan Štefane, Helena Brodnik, Mario Trifkovič, Ivan Kvasić, Erik Zupanič, Igor Muševič

**Affiliations:** 1 Jožef Stefan Institute, Jamova 39, 1000 Ljubljana, Slovenia; marion.van.midden@ijs.si (M.v.M.); matija.koterle@student.fmf.uni-lj.si (M.K.); vid.prezelj@student.fmf.uni-lj.si (V.P.); ivan.kvasic@ijs.si (I.K.); erik.zupanic@ijs.si (E.Z.); igor.musevic@ijs.si (I.M.); 2 Faculty of Electrical Engineering, University of Ljubljana, EE dep., Tržaška 25, 1000 Ljubljana, Slovenia; drago.strle@fe.uni-lj.si (D.S.); mario.trifkovic@fe.uni-lj.si (M.T.); 3 Faculty of Chemistry and Chemical Technology, University of Ljubljana, Večna pot 113, 1000 Ljubljana, Slovenia; bogdan.stefane@fkkt.uni-lj.si (B.Š.); Helena.Brodnik@fkkt.uni-lj.si (H.B.); 4 Faculty of Mathematics and Physics, University of Ljubljana, Jadranska 19, 1000 Ljubljana, Slovenia

**Keywords:** artificial nose, e-nose, electronic nose, detection of explosives, chemical selectivity of e-nose, arrays of sensors, machine learning and sensor arrays

## Abstract

We used a 16-channel e-nose demonstrator based on micro-capacitive sensors with functionalized surfaces to measure the response of 30 different sensors to the vapours from 11 different substances, including the explosives 1,3,5-trinitro-1,3,5-triazinane (RDX), 1-methyl-2,4-dinitrobenzene (DNT) and 2-methyl-1,3,5-trinitrobenzene (TNT). A classification model was developed using the Random Forest machine-learning algorithm and trained the models on a set of signals, where the concentration and flow of a selected single vapour were varied independently. It is demonstrated that our classification models are successful in recognizing the signal pattern of different sets of substances. An excellent accuracy of 96% was achieved for identifying the explosives from among the other substances. These experiments clearly demonstrate that the silane monolayers used in our sensors as receptor layers are particularly well suited to selecting and recognizing TNT and similar types of explosives from among other substances.

## 1. Introduction

The past couple of years have seen an increase in the amount of research on artificial noses for detecting targeted substances in the atmosphere. While the first generations of sensors were optimized to respond to a particular substance and were designed to detect it within a certain concentration range, sensor sensitivity is not a major problem anymore. But because these sensors can have the same electrical response to many different targeted substances, the necessary chemical selectivity can be hard to achieve. Therefore, research is now shifting towards arrays of an increasing number of sensors that are able to distinguish between a series of different substances in various concentration ranges, as a dog’s nose would. The design of a sensor array depends on the application, for example, [1] industrial chemicals, such as pollutants, volatile organic compounds, or explosives [2], or compounds used in environmental monitoring. Often, the applications are in the food and beverage industry [3,4], for detecting fruit aromas or determining the ripening status [5,6], controlling the quality of vegetable oil [7,8], classifying different types of wines [9] or teas [10] and in detecting spoilage due to microbiological contamination [11]. Applications in the field of medicine have been explored as well, for example, in analysing breath [12] or detecting the volatile organic compounds produced by bacteria in infected wounds [13]. There are a variety of technologies used in artificial noses. Some of the most common sensors are based on metal oxide semiconductors (MOS), while other types use conducting polymers or employ approaches from optics, mass spectrometry, gas chromatography, or combinations of techniques [4]. For several applications in food chemistry, a small number of highly sensitive sensors is already sufficient, as we can deduce the state of the sample based on the presence of a small number of compounds [4]. However, the fundamental question remains, whether a more general system can be built, one that truly mimics a dog’s nose. The long-term vision is an array of thousands of different sensors, integrated onto a single chip, similar to image sensors. Such a system with many sensors that selectively respond to different substances would make the detection of a wider set of substances possible. In addition, such a system would allow us to simultaneously test several different surface modifications, which would mean the faster optimization of an e-nose with a small number of sensors for specific applications. It is important to note that the present system of surface-functionalized micro-capacitors allows for a wide variety of organic receptor molecules to be designed for specific sensing applications.

To date, the number of different sensors in an array is small. The maximum number of different sensors reported in the literature is 18 semiconductor sensors installed in a commercially available e-nose [10] or 34 in an experimental setup [13]. We have built a 16-channel e-nose demonstrator [2] for use in this study. The number of sensors in existing e-noses is small compared to the millions of sensing cells in a dog’s nose. But while increasing the number of sensors is a plausible task and a large number of sensors could be integrated on a chip, similar to CMOS chips for imaging, the real problem is handling, analysing and interpreting the huge amounts of data that are generated from such a sensor array.

Due to the complexity of a sensor array’s output, one option to handle the data is the use of artificial intelligence (AI). The past couple of decades have witnessed remarkable advances in the capabilities of AI to manage large amounts of data in very different scientific fields. AI is successfully used where “big data” is generated, such as in particle physics, astronomy, molecular biology and medicine. 

In order to properly interpret the “signal” from a multisensory array, careful data analysis is required. Often, machine-learning methods are employed to help with this task. Typically, the analysis consists of data pre-processing, feature extraction, building classification models, and decision making, which means classifying the input to the correct class. As the sensory system is aimed to tackle a particular task, the methods have to be optimized for the domain in question. Data pre-processing usually removes the corrupted data, filters, segments, and assigns classes to the segments. Feature extraction aims to extract robust information from the sensors’ responses, with common features being the average signal differences, the relative differences, or the different types of array normalizations [14]. The classification methods can be seen as unsupervised and supervised. Unsupervised methods work on data that have not been labelled and aim to identify commonalities. They usually work by building clusters of data based on the statistical properties. A commonly used technique with unsupervised methods is principal component analysis (PCA) [15]. On the other hand, supervised methods require both a training dataset, upon which the model is built, as well as a testing set, upon which it is evaluated. Here, both sets are labelled, meaning that the samples are assigned to chosen classes (such as individual chemical compounds). Some successful methods applied to electronic noses include support vector machines (SVMs) [5,12], different types of neural networks [9,12,16], as well as different methods based on decision trees. Overall, machine-learning methods usually perform well when assisting with the classification of sensor inputs. Some other algorithm-related tasks with electronic noses include the compensation of sensor drift, an intrinsic feature of the sensor that appears with time [17], developing approaches to incrementally add classes to the model without having to retrain it for each new class [18] and knowledge transfer between similar sensors [19].

The goal of this study is to apply the methods of artificial intelligence to an existing e-nose demonstrator, which is described in our earlier publication [2]. There are 16 different pairs of sensors in this demonstrator, which are differently chemically surface-functionalized, thereby providing different electrical responses to different vapours. We selected 11 different target substances, which are presented in Table 1. With many sensors and many different compounds, we want to see which sensors or sensor combinations are appropriate for particular compounds.

Our approach is to measure the response of each sensor in the array to each targeted substance at different concentrations of vapours and different flow rates, which allows us to gather more data on a single substance in a controlled manner. As a result, we obtain a 2D array of responses for each sensor at different concentrations and flow rates for each targeted substance. For each sensor, this amounts to 50 different responses (all functions of time) for every selected substance. From the stored functional responses, we assign characteristic parameters, e.g., the amplitude of a response for a certain combination of flow rate and concentration, which is a single number. These numbers are then organized into a matrix that is presented with a so-called “heat map”. Such maps give a very informative overview of the response of a certain sensor to a selected substance. As our demonstrator has 16 channels, we obtain 16 such matrices for every substance with 16 × 50 = 800 numbers characterizing the response of the sensor array to that substance.

It has been previously demonstrated that many of the sensors in our demonstrator system react to various substances in a different way; however, several sensors also show a response to more than one substance, which makes the interpretation of the signals non-trivial. Since we are dealing with a new type of sensor, we only use AI methods that are easy to interpret in order to understand which features are the most relevant to differentiating between individual substances. In this paper we explore the use of the Random Forest machine-learning algorithm to distinguish the array responses to different substances. After the algorithm is trained on a set of acquired signals, the question is how well the algorithm recognizes newly acquired signals from one of the substances of the set. At this point we are only interested in identifying individual, “pure” substances, with future work planned to look at mixtures of different vapours.

## 2. Materials and Methods

### 2.1. Array of Sensors

An array of 16 micro-capacitive sensors that were chemically functionalized using different receptor molecules was used [2]. Each sensor is actually a chip, which has a pair of identical, planar, comb-like micro-capacitors with inter-digitated electrodes made of a thin layer of silicon dioxide. Each micro-capacitor has outer dimensions of 350 µm × 300 µm and has 51 fingers, each finger is 300 µm long. The electrodes are made of polysilicon and are 1.5 µm apart and 2.5 µm high. The conductive polysilicon is covered with an approximately 10-nm-thick layer of SiO_2_. This layer provides for the good chemical binding of different organic molecules, which serve as a thin receptor layer to attract targeted molecules in the surrounding atmosphere and bind them to the surface. This surface layer of attracted molecules changes the capacitance of the sensor, which is detected by our electronic circuit. After the chip and the pair of micro-capacitors is surface functionalized with different receptor molecules, the receptor layer of one of the micro-capacitors is removed using high-intensity Ar^+^ laser illumination. This illumination produces a high surface temperature and the illuminated receptor layer loses its ability to preferentially attract targeted molecules to that surface. In this way the pair becomes chemically different and the preferential adsorption of the targeted molecules on one sensor causes an imbalance in the capacity of the two sensors. This imbalance is then detected as the signal from each sensor pair.

The surfaces of the 15 sensors used in this system were modified with six different silanes: (1) 3-aminopropyl) trimethoxysilane (APTMS), (2) *p*-aminophenyltrimethoxysilane (APhS), (3) 1-[3-(trimethoxysilyl)propyl] urea (UPS), (4) *N*-(2-aminoethyl)-3-aminopropyltrimethoxysilane (EDA), (5) *N*,*N*-dimethylaminopropyl)trimethoxysilane (DMS) and (6) octadecyltrimethoxysilane (ODS). After the comb-like micro-capacitors were coated with a specific silane, one of the micro-capacitors in each pair was irradiated with a high-power laser beam to modify the properties of the organic layer. In many cases this irradiated sensor showed different responses than the non-treated and was considered as an independent sensor in our measurements. This means that we actually had 30 different sensors operating in our e-nose demonstrator. The processes of surface modification by these organic molecules and the characterization of the surfaces is comprehensively described in Ref. [2].

### 2.2. 16 Channel e-Nose Demonstrator

**Figure 1:**
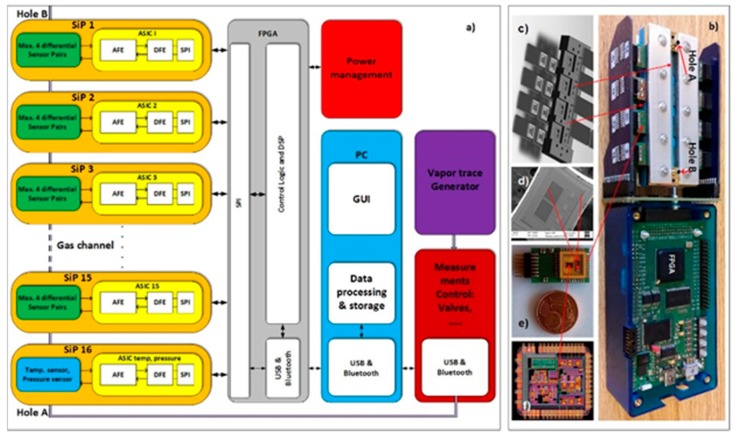
16-channel e-nose demonstrator based on micro-capacitors. (**a**) Block diagram of the 16-channel e-nose. (**b**) Physical implementation of the e-nose. (**c**) Holder for individual sensor PCBs, which is made of thin sheets of low-temperature ceramics, stacked together to form a 3D structure with voids and channels where the air is pumped through. (**d**) SEM image of a single chip with two comb micro-capacitors, (**e**) System in package, (**f**) Layout of the ASIC.

A block diagram of the 16-channel e-nose demonstrator for vapour-trace detection is presented in Figure 1a. It is composed of 16 differently modified comb capacitive sensors connected to an ASIC with detection electronics. One channel of detection electronics can serve a maximum of four differential sensors. In this experiment we used only two sensors in one channel of electronics. The result of the capacitance-difference measurement of each sensor is A/D converted and further processed in the FPGA [2]. The last, 16th channel serves for the temperature and humidity measurements. The results are sent to a PC for further processing, storage, eventual display and further signal processing using the methods of machine learning, as described here. The physical implementation of the 16-channel e-nose demonstrator is presented in Figure 1b, while its building blocks are presented in Figure 1c,d. The technical details of the demonstrator are explained in the references [2,20,21].

**Figure 2:**
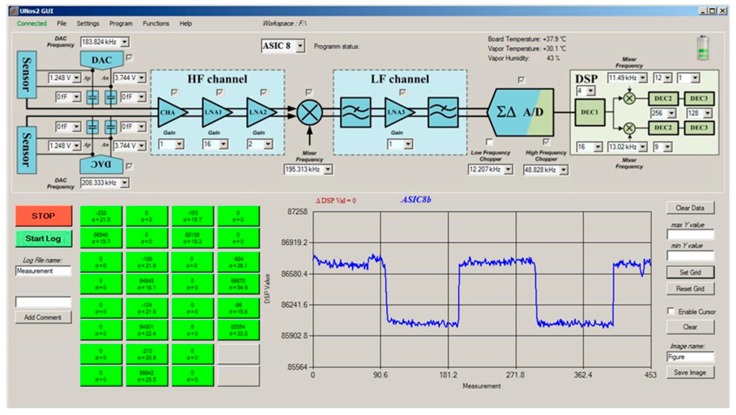
PC interface screen of the 16-channel e-nose.

Figure 2 shows a PC interface screen of the 16-channel e-nose demonstrator, where the response of each sensor can be plotted vs. time at a rate of 100 points/minute. At the same time, each sensor’s response is analysed and plotted as a coloured square with a colour corresponding to the magnitude of the response. In this way a matrix of coloured squares is presented, as shown on the bottom-left part of Figure 2, which is helpful for monitoring and comparing the responses of different sensors. In the figure all the squares are green because no thresholds for the measured signal have been defined.

### 2.3. Generator of Vapours and Measuring Protocols

To measure the response of the 16-channel e-nose demonstrator to different vapours we need different vapours with adjustable and calibrated concentrations for each vapour. We have previously published detailed studies of a reliable generator of vapours for different explosives; this is an important instrument for the development of e-noses [2,20,21]. We used two different vapour generators in this study. For explosives, the setup for generating particular concentrations of molecules of different explosives in the N_2_ carrier gas is based on the flow of N_2_ through containers with finely dispersed explosives on a fibre carrier, which provides a large surface area of explosive material [2,20,21]. For the remaining substances, gas samples of high purity were prepared commercially by mixing the target substance with nitrogen gas to a selected concentration. These were further mixed with additional N_2_ carrier gas to lower the concentration to the required value at a certain total flow and fed into the measuring system. It was noticed in our experiments that humidity fluctuations in the laboratory significantly influenced the sensors’ responses. For this reason, the e-nose demonstrator was enclosed in a metal tube, which had an overpressure of N_2_ to minimise any diffusion of water into the system, thereby providing a stable environment. To prevent water diffusing through the exhaust tube, a long exhaust tube with a drying stage and a pump were used.

### 2.4. Data Acquisition

In total, 11 different chemical substances were used for the measurements, as listed in Table 1. All the targeted substances were detected in their vapour phase, which was a mixture of known concentrations of molecules of the targeted substance and the carrier gas. Very pure N_2_ was as the carrier gas because it is inert and does not interact with the sensors.

For each target substance, we varied both the concentration of the substance and the gas flow during the measurements to increase the diversity of the available dataset in a controlled manner. The concentration was varied by changing the ratio of the sample-containing gas and the pure nitrogen gas, ranging from 10% (one part of sample-containing gas and nine parts of pure nitrogen) to 100% (sample-containing gas only) in steps of 10%. The total gas flows ranged from 5 to 25 mL/min in steps of 5 mL/min and in all cases we observed that the amplitude of the sensors’ responses after a selected time period seemed to depend on the flow rate. The simplest explanation for this behaviour is that small flow rates do not allow the specified concentration to be reached in the pre-set time interval, resulting in a smaller concentration at individual sensor sites and therefore smaller responses. Nevertheless, the use of different flow rates gives us a way to gather more data in a controlled manner. Based on our observations, we only used data gathered at higher flow rates in our final analyses.

For a given concentration and flow rate the measurement started by flushing the sensor array with pure N_2_ for 5 min, in the case of explosives, and 3 min, in the case of the other substances. This is a “cleaning cycle”, or the “off” cycle, where the remaining molecules, adsorbed on the surfaces of the micro-capacitive sensors and inside the connecting tubes, were removed by the N_2_. Then, the gas with a chosen concentration flowed through the sensor array for 3 or 5 min, depending on the substance. This is the “on” cycle, which together with the “off” cycle, form a complete cycle. This cycle was repeated 10 times. Afterwards, the array was flushed with nitrogen and the measurement continued for a different combination of flow and concentration.

In order to avoid possible bias due to the slowly shifting background from the water molecules present in the sensor system due to their diffusion from the environment, the combinations of concentration and flow were chosen in a random sequence instead of a gradual one. For explosives, the entire sequence was repeated several times. In addition, subsequent measurements took place after a few weeks or even months. On the other hand, due to the limited number of gas mixtures, the gas samples were measured only once, in some cases with a smaller number of combinations.

### 2.5. Data Processing and Feature Extraction

**Figure 3:**
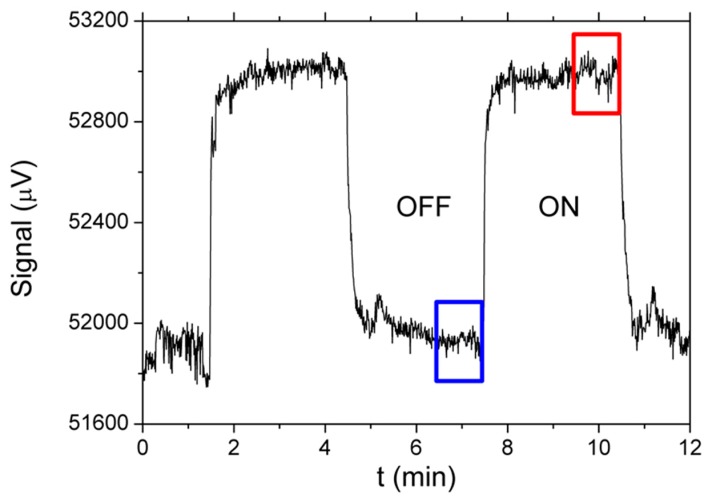
Typical section of measured time dependence of a signal for a chosen sensor (black line). Blue rectangle indicates the last part of the signal in the “off” state (bottom). Red rectangle indicates the last part (steady state) of the response to the sample in the “on” state (top). Both parts together form one row in the segment matrix.

Since machine-learning models are usually not built using raw data, such as the recorded time-dependence of a signal from each micro-capacitive sensor, we first process the data to extract meaningful information that will help us distinguish between the different substances. A typical response of an individual sensor from the array for a couple of “on” and “off” states is shown in Figure 3. When the sensor is exposed to the flow of pure N_2_, it takes approximately τ ~ 3 min to reach the steady, “off” state. This time constant τ is determined by the time required for the whole of the tubing and the chamber(s), where the sensors are mounted, to be filled with pure N_2_. Should the system be integrated, the response time would be much shorter.

When the target sample-containing gas is introduced into the chamber where the sensor is located, the signal increases to a steady value, which we call the “on” state. When the valve with the sample-containing gas is closed and only pure nitrogen gas flows over the sensor again, the signal returns to the signal value in the “off” state. The signal value in the “off” state is subjected to long-term drifts within the time window of the experiment and has to be set before the measurement begins. Therefore, at the beginning of the measurements we manually adjust this “off” signal within the dynamic range of the detecting electronics to prevent saturation effects when the drift drives the signal to the limits of the electronics.

The difference between the “on” and “off” signals is called the amplitude of the response and is determined automatically for a stored signal using an appropriate algorithm. From the stored signal, we create the *segments* that will be used for building and testing the models in the following way: first, we take 100 points of baseline immediately before the opening of the valve (i.e., the “off” state) with the sample-containing gas and the last 100 points of the response to the sample, just before switching back to pure nitrogen (the “on” state, as indicated in Figure 3). For each sensor and for a single on/off cycle, this means one row with 200 numbers, and since we have 31 sensors (30 functionalized sensors and one for humidity), each *single-cycle segment matrix* will consist of 31 rows with 200 numbers. For each measurement cycle on a selected sample, covering all the concentrations and all the flows, generates 10 concentrations × 5 flow rates × 10 repetitions, which equals 500 segment matrices. We should note that the time constant τ related to the transient effects cannot be used to distinguish between different substances, because in our system τ is primarily determined by the design of the chambers where the sensors are installed, the length of the tubing used to supply the gas, and the flow rate of the N_2_ carrier gas through the sensing head.

The next step is called *feature extraction* in machine-learning terminology. Each feature is a mathematical operation upon the segment matrix, which produces a single numerical value and is explained in the continuation. For each segment matrix, we calculate a series of features. The values are then stored in a vector that is called an instance. In a machine-learning approach, it is common to generate a large number of features, especially when it is not immediately clear which features will be the most efficient for classification. In our case, we calculated the following five features:

- The amplitude for each functionalized sensor (the difference between the “on” and “off” signals, which makes 30 features in total).
- The noise difference for each sensor. We calculated the RMS noise in the “on” state and subtracted the RMS noise in the “off” state. This feature is not a “very strong” one, as it is not much different for different sensors and shows rather random behaviour in the noise heatmaps.
- The flow rate. We use the flow-rate value as a feature as we can determine or set it independently. At the same time, we keep the concentration as an unknown variable. This choice can be rationalized if we consider the operation of the algorithm in a real situation with a real demonstrator. If we are seeking for a specific substance, then we do not know the concentration of that substance in the measuring air, but we do know the flow rate of the air entering the detector.
- The flow-normalized amplitude, because the amplitude increases with an increasing flow rate.
- The amplitude with the subtracted amplitude of the humidity sensor. This is always a positive number and will compensate the signal for possible changes to the humidity during the course of the measurements.

In total, this leaves us with 4 × 30 + 1 (flow is a single feature) = 121 features. In our measurements each instance is therefore a vector with 121 dimensions.

### 2.6. Machine-Learning and Classification

Machine-learning algorithms are used to recognize patterns in large sets of data. Often, various approaches are tested on the data to assess which performs better in terms of classification accuracy. In addition, different algorithms differ in terms of the comprehensibility to a human user. We decided to use the decision-trees algorithm (J48 algorithm), which is simple to understand. The classification of an instance begins at the root and proceeds along the branches, with each branch corresponding to a particular feature, until a leaf, corresponding to the class, which in our case is the identified substance. When building a decision tree on a training data set, features with the highest information gain are chosen first, being the features that best split the training set into distinct groups. Thus, inspecting the final decision tree provides us with an insight into which features are the most relevant for classification. However, when using decision trees, we can find ourselves in danger of overfitting the tree on the training data set. Random Forest is a further improvement to the decision-tree algorithm, which is aimed at preventing this and improving the classification accuracy. As the name suggests, the algorithm builds several decision trees, each of them on a randomly chosen subset of training data and using a randomly selected subset of features. Each instance in the testing set is then classified using all the trees in the forest and the final class is the one chosen by most trees.

Other commonly used algorithms are more complex. For example, Support Vector Machines (SVMs) looks at the data in a multidimensional space, with each dimension corresponding to a feature, and then searches for a hyperplane that best splits the data. Neural networks, which have become popular lately, consist of a complex interconnected network of “neurons”, each representing a mathematical operation on the input data, until the output represents the final class. While often very efficient, these advanced approaches essentially function as “black boxes”, with the classification process incomprehensible to human interpretation. As comprehensibility of the models was one of our main aims, only algorithms based on decision trees were used at this stage.

The classification accuracy of an algorithm is defined as the ratio of true positives (correctly classified examples, where the criteria is either YES or NO) and all the examples in the test set. To evaluate the classification accuracy of our algorithm, the data (from here on, we are only working with the instances) are split into a training set and a testing set. The training set serves for training the classification algorithm, and the testing set serves for testing the classification accuracy of the “as-trained algorithm”. To avoid overfitting, the training and testing sets must be distinct and uncorrelated. In our study, we obtained several runs of measurements from TNT, DNT and RDX, sometimes separated by weeks or months, as the vapour generator is essentially a limitless source for our purpose. Measurements from individual runs were then assigned to one of the sets. For samples from gas cylinders, due to the small amounts available, the measurements were carried out in a single run. The first half was assigned to the training and the second to the learning set, which still preserves sufficient diversity for our purpose.

## 3. Results

### 3.1. Responses of Individual Sensors

**Figure 4:**
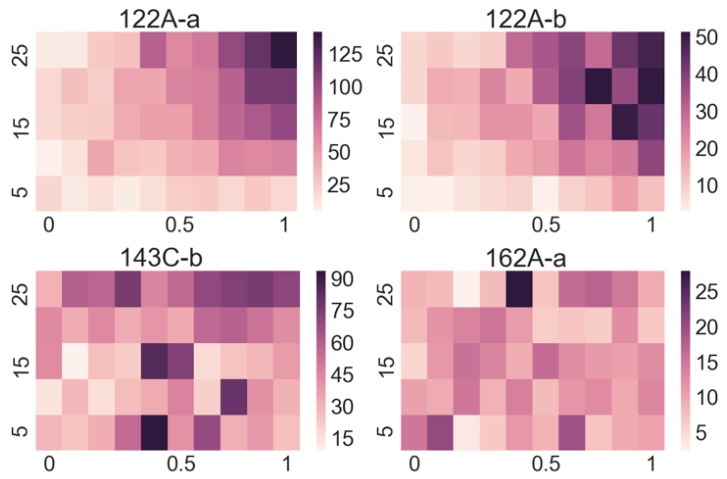
Signal amplitudes for four sensors in response to butane. The *x*-axis shows the concentration of the target substance (here, butane), normalized to the value in Table 1. The *y*-axis corresponds to flow rate in units of mL/min. The two sensors 122A-a and 122A-b in top row show good responses. The amplitude is monotonously increasing with the concentration and the flow rate. The two sensors 143C-b and 162A-a in the bottom row are not responding systematically. Note: individual sensors can have substantially different responses, which is why the colour scheme is adapted to and is unique to each heat map for clarity. 122A-b is modified with *p*-aminophenyl)trimethoxysilane (APhS), 162A-b and 163A-b with octadecyltrimethoxysilane (ODS), 132B-a with 1-[3-(trimethoxysilyl)-propyl]urea (UPS).

**Figure 5:**
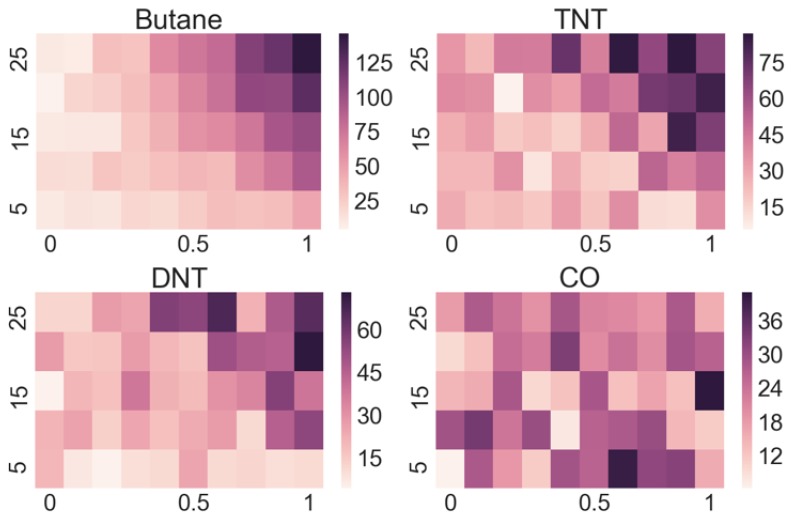
Comparison of responses of sensor 122A-a to butane, TNT, DNT and CO. This sensor shows a strong systematic response to butane, somehow weaker and noisy, but still a good response to both TNT and DNT, and a weak and a rather random response to CO.

A very useful method to assess how prominently individual sensors respond to particular substances is to plot the so-called heat maps, where we plot the amplitudes as a function of flow and concentration. To reduce the experimental noise, the amplitudes of all 10 repetitions at a single concentration and flow were averaged for the purpose of the plot. From such plots, we can easily see if the signal amplitude increases with an increasing flow and/or concentration (which means that the sensor is useful to detect a particular substance) or does not respond to it, as illustrated in Figure 4 for the responses to butane. On the other hand, we also notice that some sensors have similar responses to more than one substance, but not to others, as illustrated in Figure 5. Both figures show the responses of the sensor 122A-a to butane (top-left image), but (intentionally) from two different measurement runs, to show the reproducibility of the measurements.

### 3.2. Classification Results

As Figure 4 and Figure 5 illustrate, individual sensors often show similar responses to different substances, while other sensors do not respond. Therefore, manually determining the substance from readings of 30 sensors is an impossible task, especially when looking only at the data for a single chosen flow and concentration, and considering the experimental noise. However, we may view the responses of all 30 sensors to each substance as a unique fingerprint.

Several “experiments” were run on the dataset, with various aims. The dataset was split into an independent training set, used to build the classification algorithms, and a testing set, used to evaluate the classification accuracy. Each instance (the vector containing 121 features, corresponding to each segment, as described above) varied three parameters, i.e., substance, concentration and flow, while only “substance” was used as a class. The classification accuracy for a chosen algorithm is defined as the number of correctly classified instances (true positives) divided by the total number of instances in the testing set. Another relevant metric to consider is the comparison with the “baseline accuracy” (as if we were randomly classifying each instance), for which we consider the majority class. This is especially relevant when dealing with datasets where the classes in the testing set are not of the same size. To illustrate this, if we have five classes of equal size, the “baseline accuracy” is 20%. On the other hand, when we have two classes with instances in a 7:3 ratio, the baseline accuracy increases to 70%. In our testing data, the number of instances for explosives was typically higher than those for gases, as the number of gases was limited.

An informative way to look at the classification results is a confusion matrix, which shows the performance of the algorithm. For example, in Table 2, the first row illustrates the situation for how well the algorithm classifies butane. The value of an element of the classification matrix does not represent the probability in the statistical sense. For example, the value of 0.81 in the classification Table 2 represents the ratio of correctly classified instances for butane. The values in the diagonal represent the correctly classified instances (correct substances determined by the row name), while the off-diagonal elements represent misclassifications as the wrong substances (columns). From the confusion matrix we can learn whether the algorithm works particularly well on some classes or which are the classes it frequently confuses. The values in the confusion matrix are normalized by rows, thus the sums of each row equal 1. The elements of the confusion matrices are presented as follows: the diagonal elements, which are close to 1, are coloured in green, while the larger off-diagonal elements are red, for easier interpretation.

In the analysis, we first make some considerations. First, we notice that our sensor array responds poorly to H_2_S and SO_2_, and we therefore exclude them from any further analysis. Second, we notice from the confusion matrices that the classification algorithms often mix TNT and DNT, since they have similar sensor responses (as hinted in Figure 5). The likely reason for this is that both molecules are very similar from the chemical point of view. In the following analysis, we therefore group both substances into a single class, provisionally called XNT.

We noticed that the instances that become misclassified are most often those measured at low concentrations and low flows, where the signal amplitudes are consequently lower and more prone to experimental noise. To build the final classification models, we therefore only kept the part of the data with the highest concentrations and flows, namely the top-three concentrations and the top-two flows. In effect, this makes the flow-related features almost irrelevant.

Table 2 and Table 3 show confusion matrices for some selected sets of substances to illustrate the classification results. Table 2 shows the result for seven classes, where we used XNT as a group containing both TNT and DNT. The overall classification accuracy using the RandomForest algorithm is 72%, with a baseline accuracy of 27% (if we classify all instances as the class with the largest number of instances). Inspecting the confusion matrix, we notice that the classification accuracy is better for some substances than for others. For methane (CH_4_) and NO, the algorithm classifies correctly more than 90% of the instances, which is an excellent result. For other substances, the accuracy is lower. For example, CO is often mistaken for ammonia (NH_3_), while ammonia itself is in almost a third of the cases mistaken for XNT.

In the next step we narrow the number of targeted substances to four, which are very well distinguished by our demonstrator and algorithm. The confusion matrix for these special cases is shown in Table 3. Here, the classification accuracy using Random Forest is 92%, with a baseline accuracy of 43%. Here, all the instances of XNT and NO were classified correctly, while some cases of CO were classified as butane or NO and 10% of butane instances were classified as XNT.

In the third example we look back at the initial development stages of our e-nose setup, which was conceived as a detector for explosives. In this test we group the substances in two groups: one contains TNT, DNT and RDX, and the other one contains the substances. Table 4 shows the confusion matrix for a binary classifier, explosives vs. other substances. The classification accuracy here is 96% (with a baseline of 61%), which is high, considering the rather heterogeneous composition of each of the two groups. The value of 0.94 for explosives in the classification Table 4 represents the ratio of correctly classified instances for explosive versus all other substances.

## 4. Discussion

Looking at the heatmaps for the individual sensor responses when varying the concentration and flow of the target substance can give us a quick insight into which sensors are appropriate for the detection of particular substances and which ones do not respond. However, as the output of a multi-sensor e-nose is complex, meaning that some sensors respond to several substances and many sensors respond to a particular substance, a manual interpretation is practically impossible.

On the other hand, methods of artificial intelligence are well suited to help us with the classification task. Apart from building the classification model, in our case using Random Forest, we can learn several things about the task by looking at the confusion matrices that have been obtained. In our case, we noticed among the studied substances that TNT and DNT are often misclassified as one another. This is most likely due to the similar chemical structure and the similar affinity of the functionalized surfaces of our sensors to these two substances. However, if we group TND and DNT together (which is reasonable due to their chemical similarity), they are well distinguished from all the other substances. The value of 0.94 for explosives in the classification Table 4 represents the ratio of correctly classified instances for explosive versus all other substances and is considered a very good result.

This leads to the conclusion that the set of six different silane molecules: (1) 3-aminopropyl) trimethoxysilane (APTMS), (2) *p*-aminophenyltrimethoxysilane (APhS), (3) 1-[3-(trimethoxysilyl)-propyl] urea (UPS), (4) *N*-(2-aminoethyl)-3-aminopropyltri-methoxysilane (EDA), (5) *N*,*N*-dimethyl-aminopropyl)trimethoxysilane (DMS) and (6) octadecyltrimethoxysilane (ODS) are very appropriate for the selective detection of TNT and DNT molecules in the atmosphere.

**Figure 6:**
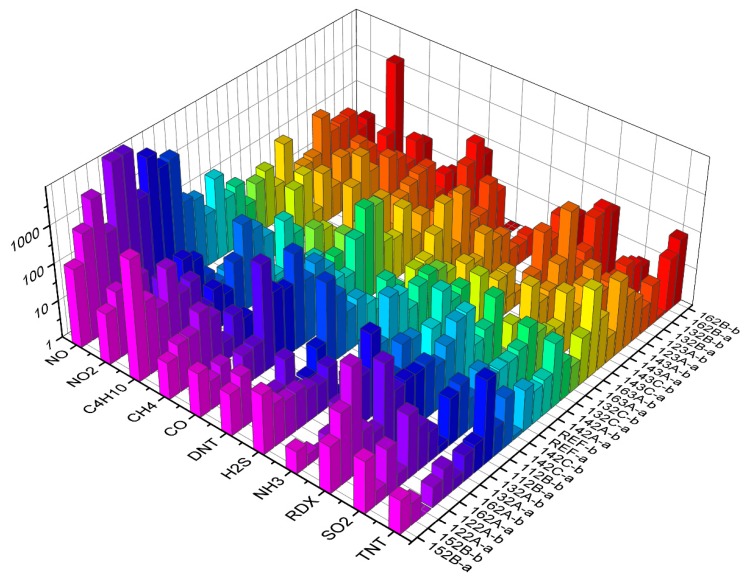
Responses of all sensors to all substances, using the signal values at maximum concentration and flow rates.

Building classification algorithms can provide an insight into which sets of substances can be distinguished well among themselves, as demonstrated in Table 2, Table 3 and Table 4. In addition, models based on decision trees are easy to interpret, allowing us to see which features are more important and which are not—if features related to a particular sensor do not appear in decision trees, the sensor can be discarded altogether when optimizing the sensor array for a particular application. For example, in the case where we distinguish explosives from other substances, an inspection of some decision trees generated by the algorithm shows that sensors REF-B, 143C-a and 132A-b play the most prominent role in the classification (they appear at the initial nodes of several trees). Looking at the responses of all the sensors to all the substances at the maximum concentration and flow (Figure 6), this is reasonable, since these sensors respond to TNT, DNT and RDX better than to some other substances.

One of the key steps in improving the classification accuracy of our algorithm was to focus only on the subset of the data with stronger signals, i.e., the measurements with higher concentrations and flows. While this might appear to be a drawback, we should remember that the initial concentration (especially for explosives) was already very low—increasing the concentration to higher values would likely result in a much higher response, making the classification task easier.

From the algorithm point of view, there are several ways to improve the accuracy, such as including additional features that are calculated as combinations of more than one sensor, or using advanced methods such as neural networks. However, as we already pointed out at the beginning, the goal was to demonstrate the feasibility of the approach and to see how machine-learning can guide us to improve and optimize the sensor array itself. Neural networks were previously used in e-noses [3,5,8,10]; however, as they are essentially “black boxes” that only produce results without an intuitive interpretation, they are only suitable for a “final” application and not for our case, where we are still in the development and optimization phase. The main takeaway from the experiments should then be seen as an insight into how to assemble sensors to work well on a chosen set of substances—which sensors to keep and whether we should add additional sensors that react to substances to which the current system responds poorly (as seen with H_2_S and SO_2_ in our case). Alternatively, if we want to distinguish between TNT and DNT, we should focus on a sensor functionalization that will exploit the difference between the two.

There are two important problems to consider for future work from the computer-science point of view. First, let us imagine a situation where the system is trained to work on a specific set of substances. Now, we want to expand the functionality of the system to work on additional classes, but we do not have the original training set (perhaps we are an end user and the manufacturer is unwilling to share the data) that would allow us to straightforwardly train the new model. Second, due to the nature of the manufacturing of individual sensors, which includes coating with a functionalized layer followed by laser abrasion, no two sensors of the same type will be identical—thus the classification model trained on the data obtained on one setup might not work with the same accuracy on another one. The task here is to optimize the classification model to work on the second setup without having to repeat all the measurements for the training set. In computer science, both of these two problems can be viewed as tasks for the transfer-learning domain [23].

## 5. Conclusions

In this paper we demonstrated that we can apply relatively simple machine-learning models to assist in the classification of sensor-array responses to various chemical substances, where we are independently varying both the concentration of the target substance in the nitrogen carrier gas and the gas flow. Classification models were built using the Random Forest algorithm, which builds a series of decision trees on subsets of the training set. Inspection of the individual decision trees can provide an insight into which sensors are particularly useful for a specific task, thus allowing us to optimize the setup if needed. The classification accuracy depends on the number of substances we want to distinguish—for seven substances, the accuracy is 72%, for the four best (including a joint class of TNT and DNT), the accuracy rises to 90%, while for a binary classifier distinguishing between explosives and other substances in the set, it reaches 96%. The experiments clearly demonstrate that the silane monolayers used in our sensors are particularly well suited to the detection of explosives. Future work will include 64 sensors with different coatings, providing sensitivity to other compounds, measuring additional sets of substances, and the simultaneous detection of combinations of two or more substances.

## Figures and Tables

**Table 1:** List of substances and their concentrations used in this study. The concentration is given in the number of molecules of the substance per one million molecules of N_2_ carrier gas. The values for TNT, DNT and RDX are calculated using the vapour pressures [22].

Substance	Chemical Formula/Name	Concentration (ppmv)	Source
Butane	CH_3_-CH_2_-CH_2_-CH_3_	79,800	Gas cylinder
Methane	CH_4_	999,950	Gas cylinder
Carbon monoxide	CO	299	Gas cylinder
Sulphur dioxide	SO_2_	13.2	Gas cylinder
Hydrogen sulphide	H_2_S	94.5	Gas cylinder
Ammonium	NH_3_	200	Gas cylinder
Nitrogen dioxide	NO_2_	15.9	Gas cylinder
Nitric oxide	NO	116.3	Gas cylinder
RDX	1,3,5-Trinitro-1,3,5-triazinane	0.00000485	Vapour generator
DNT	1-Methyl-2,4-dinitrobenzene	0.4	Vapour generator
TNT	2-Methyl-1,3,5-trinitrobenzene	0.00915	Vapour generator

**Table 2:** Confusion matrix for seven classes, using the Random Forest algorithm. Red and green colours are used to highlight some of the most relevant elements.

%	Butane	CH_4_	CO	XNT	NH_3_	NO	NO_2_
Butane	0.81	0	0.06	0	0.13	0	0
CH_4_	0.08	0.92	0	0	0	0	0
CO	0	0	0.44	0	0.5	0	0.06
XNT	0	0	0	0.63	0.08	0.08	0.21
NH_3_	0	0	0	0.31	0.63	0.06	0
NO	0	0	0	0	0.06	0.94	0
NO_2_	0	0	0	0.38	0.19	0	0.43

**Table 3:** Confusion matrix for four classes, using the Random Forest algorithm.

%	Butane	CO	XNT	NO
Butane	0.9	0	0.1	0
CO	0.13	0.8	0	0.04
XNT	0	0	1	0
NO	0	0	0	1

**Table 4:** Confusion matrix for explosives vs. other substances using the Random Forest algorithm.

%	Explosives	Other
Explosives	0.94	0.06
Other	0.03	0.97

## References

[1] Hsieh Y.C., Yao D.J. (2018). Intelligent gas-sensing systems and their applications. J. Micromechanics Microengineering.

[2] Strle D., Štefane B., Trifkovič M., Van Miden M., Kvasić I., Zupanič E., Muševič I. (2017). Chemical selectivity and sensitivity of a 16-channel electronic nose for trace vapour detection. Sensors.

[3] Loutfi A., Coradeschi S., Mani G.K., Shankar P., Rayappan J.B.B. (2015). Electronic noses for food quality: A review. J. Food Eng..

[4] Röck F., Barsan N., Weimar U. (2008). Electronic nose: Current status and future trends. Chem. Rev..

[5] Qiu S., Wang J., Tang C., Du D. (2015). Comparison of ELM, RF, and SVM on E-nose and E-tongue to trace the quality status of mandarin (*Citrus unshiu* Marc.). J. Food Eng..

[6] Adak M.F., Yumusak N. (2016). Classification of E-nose aroma data of four fruit types by ABC-based neural network. Sensors.

[7] Bougrini M., Tahri K., Haddi Z., Saidi T., El Bari N., Bouchikhi B. (2014). Detection of adulteration in argan oil by using an electronic nose and a voltammetric electronic tongue. J. Sens..

[8] Majchrzak T., Wojnowski W., Dymerski T., Gębicki J., Namieśnik J. (2018). Electronic noses in classification and quality control of edible oils: A review. Food Chem..

[9] Aguilera T., Lozano J., Paredes J.A., Alvarez F.J., Suárez J.I. (2012). Electronic nose based on independent component analysis combined with partial least squares and artificial neural networks for wine prediction. Sensors.

[10] Pławiak P., Maziarz W. (2014). Classification of tea specimens using novel hybrid artificial intelligence methods. Sens. Actuators B Chem..

[11] Falasconi M., Concina I., Gobbi E., Sberveglieri V., Pulvirenti A., Sberveglieri G. (2012). Electronic nose for microbiological quality control of food products. Int. J. Electrochem..

[12] Leopold J.H., Bos L.D., Sterk P.J., Schultz M.J., Fens N., Horvath I., Bikov A., Montuschi P., Di Natale C., Yates D.H. (2015). Comparison of classification methods in breath analysis by electronic nose. J. Breath Res..

[13] Sun H., Tian F., Liang Z., Sun T., Yu B., Yang S.X., Liu X. (2017). Sensor array optimization of electronic nose for detection of bacteria in wound infection. IEEE Trans. Ind. Electron..

[14] Yan J., Guo X., Duan S., Jia P., Wang L., Peng C., Zhang S. (2015). Electronic nose feature extraction methods: A review. Sensors.

[15] Hotel O., Poli J.P., Mer-Calfati C., Scorsone E., Saada S. (2018). A review of algorithms for SAW sensors e-nose based volatile compound identification. Sens. Actuators B Chem..

[16] Xu L., He J., Duan S., Wu X., Wang Q. (2016). Comparison of machine learning algorithms for concentration detection and prediction of formaldehyde based on electronic nose. Sens. Rev..

[17] Rehman A.U., Bermak A. (2018). Drift-Insensitive Features for Learning Artificial Olfaction in E-Nose System. IEEE Sens. J..

[18] Cheng Y., Wong K.Y., Hung K., Li W., Li Z., Zhang J. (2018). Deep Nearest Class Mean Model for Incremental Odor Classification. IEEE Trans. Instrum. Meas..

[19] Zhang L., Liu Y., Deng P. (2017). Odor recognition in multiple E-nose systems with cross-domain discriminative subspace learning. IEEE Trans. Instrum. Meas..

[20] Strle D., Stefane B., Nahtigal U., Zupanic E., Pozgan F., Kvasic I., Musevic I. (2011). Surface-functionalized COMB capacitive sensors and CMOS electronics for vapor trace detection of explosives. IEEE Sens. J..

[21] Strle D., Štefane B., Zupanič E., Trifkovič M., Maček M., Jakša G., Muševič I. (2014). Sensitivity comparison of vapor trace detection of explosives based on chemo-mechanical sensing with optical detection and capacitive sensing with electronic detection. Sensors.

[22] Ewing R.G., Waltman M.J., Atkinson D.A., Grate J.W., Hotchkiss P.J. (2013). The vapor pressures of explosives. TrAC Trends Anal. Chem..

[23] West J., Ventura D., Warnick S. (2007). Spring research presentation: A theoretical foundation for inductive transfer. Brigh. Young Univ. Coll. Phys. Math. Sci..

